# LncRNA MBNL1-AS1 represses gastric cancer progression via the TGF-β pathway by modulating miR-424-5p/Smad7 axis

**DOI:** 10.1080/21655979.2022.2037921

**Published:** 2022-03-21

**Authors:** Jiewen Su, Dawei Chen, Yi Ruan, Yuan Tian, Kaiji Lv, Xinhua Zhou, Dongjian Ying, Yeting Lu

**Affiliations:** a Department of Gastroenterology, the Affiliated Lihuili Hospital, Ningbo University, Ningbo, Zhejiang, PR China; b Department of General Surgery, the Affiliated Lihuili Hospital, Ningbo University, Ningbo, Zhejiang, PR China

**Keywords:** lncRNA MBNL1-AS1, gastric cancer, miR-424-5p, Smad7, TGF-β/EMT pathways

## Abstract

Studies over the past decades have implicated lncRNAs in promoting the development, migration and invasion of gastric cancer (GC). However, the role and mechanism of lncRNA MBNL1-AS1 in GC promotion are poorly understood. In this research, qRT-PCR showed that MBNL1-AS1 was down-regulated in GC tissues and cells. Cell experiments and the animal study demonstrated that MBNL1-AS1 knockdown accelerated GC cell proliferation, migration, and invasion, thus restraining cell apoptosis. Meanwhile, overexpression of MBNL1-AS1 repressed GC cell promotion. Bioinformatics analysis confirmed that MBNL1-AS1 binds to miR-424-5p via negative modulation. Rescue experiments showed that decreased miR-424-5p level inhibited GC cell promotion by silencing MBNL1-AS1. Furthermore, Smad7 was identified as a target of miR-424-5p that could reverse the promotion of GC cell growth mediated by miR-424-5p. Western blot results proved that MBNL1-AS1 affected TGF-β/SMAD pathways by regulating the miR-424-5p/Smad7 axis. Collectively, MBNL1-AS1 restrained GC growth via the miR-424-5p/Smad7 axis and thus could be a promising target for GC therapy. These findings illustrate that lncRNA MBNL1-AS1, as a tumor suppressor gene, participates in GC progression by regulating miR-424-5p/Smad7 axis, thus activating TGF-β/EMT pathways. The evidence may provide a potential marker for GC patients.

## Graphical abstract

**Figure:**
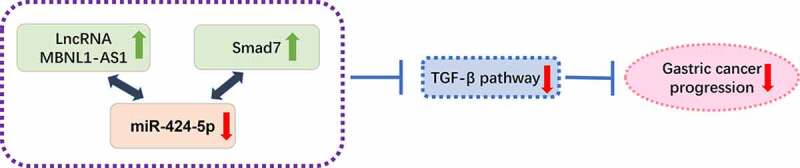

## 1. Introduction

Gastric cancer (GC) is one of the most prevalent malignant tumors in the world. The latest GLOBOCAN statistics shows that GC ranks fifth based on the incidence and the mortality rate of all malignant tumors [1]. In 2012, there were about 951,000 new cases of GC worldwide, of which more than 70% were from developing countries [2]. Nearly half of the cases occurred in East Asia [3]. According to recent statistics in China, as a country with a large population, the incidence and mortality of GC in China rank third among all malignant tumors [4]. Presently, surgery is the major treatment strategy for GC. Moreover, chemotherapy-based systemic therapy, molecular targeted drugs, and radiotherapy are other main treatments for advanced GC. Studies have shown that the median overall survival of patients with advanced GC who received combined chemotherapy is 7.6 ~ 11.6 months, with a poor prognosis [5]. Targeted therapy and immunotherapy are becoming novel strategies for GC treatment due to their rapid progress [6,7].

LncRNAs (long-stranded non-coding RNAs) are a subset of RNA transcripts with a length of more than 200 bp and cannot encode proteins [8]. Like mRNA encodes proteins, lncRNA is also transcribed and synthesized via RNA polymerase. LncRNA often exists in the cytoplasm or nucleus and has certain tissue specificity and poor conservation of interspecific sequences [9]. Except that lncRNA has low expression level, relatively few nucleotides, and a few longer exons, many other aspects are similar to the sequence of mRNA [10], including 5’-cap structure, 3’- polyadenylate tail structure, intron, and splice site. Many studies have shown that lncRNA, as a carcinogenic or tumor suppressor gene, is involved in the occurrence and development of tumors [11]. Many lncRNAs are diversely expressed in liver cancer, lung cancer, and other tumor cells. Therefore, they could become a new tumor biomarker and therapeutic target for tumor diagnosis, treatment, and prognosis monitoring [12–14].

Studies have provided important information on lncRNAs participating in GC promotion over the past decades. For instance, lncRNA GACAT3 is up-regulated in GC and may act as an oncogene to promote GC progression. Further functional tests have proved that miRNA-497 can directly bind to GACAT3 and down-regulate its expression level, thus influencing GC cell progression [15]. Additionally, lncRNA ADAMTS9-AS2 is significantly down-regulated in GC tissues and cells, thus inhibiting the life cycle of GC cells and inducing apoptosis by activating PI3K/Akt pathway [16]. LncRNA MALAT1/ miR-30e/ ATG5 regulatory axis can also promote drug resistance of GC cells to DDP. Most lncRNAs and miRNAs were reported that can participate in cell autophagy [17,18]. LncRNA MALAT1 participates in the autophagy of GC cells by binding to miR-30e and regulating the expression of ATG5. Silencing MALAT1 can inhibit chemically-induced autophagy and enhance the sensitivity of GC cells to DDP [19]. Therefore, studying the differentially expressed lncRNA in GC can determine new molecular markers for GC.

Only a few studies have assessed the function of lncRNA muscle blind-like 1 antisense RNA 1 (MBNL1-AS1). MBNL1-AS1 is signally-declined in NSCLC and inhibits tumor progression by binding with miR-135a-5p [20]. Furthermore, MBNL1-AS1 can function as a sponge to modulate miR-362-5p level in bladder tumor growth [21]. However, the role of MBNL1-AS1 in GC is unknown.

Herein, this study is aimed at disclosing the specific function and underlying mechanism of lncRNA MBNL1-AS1 in GC. The exploration of MBNL1-AS1ʹs network may provide effective strategies for GC.

## 2. Methods

### 2.1 Patient tissues

In this study, 60 pairs of GC tissues and adjacent normal tissues were obtained from the Department of General Surgery, Ningbo Medical Center, Lihuili Hospital, between January 2015 to December 2015. The whole tissues were immediately dissected and frozen in liquid nitrogen. Two or more pathologists confirmed the specimens in the hospital. The Ethics Committee of Lihuili Hospital approved this research, and all patients signed informed consent forms.

### 2.2 Bioinformatics prediction and prognostic analyses from TCGA database

TCGA (https://www.cancer.gov/) is a widely used online database for integrated investigation. Herein, the bioinformatics forecast of GC was conducted using the TCGA database. Threshold values were set at p < 0.05 and |log_2_ FC| ≥ 1.

### 2.3 Cell culture and cell transfection

Five GC cell lines (AGS, MGC803, BGC-823, SGC-7901, HGC-27) and human gastric mucosal epithelial cell line GES-1 were cultured in 1640 medium with 10% FBS and 1% antibiotics at 37°C and 5% CO_2_. The AGS cells in the logarithmic growth period were then seeded in a six-well plate (2 × 10^5^ cells per well). Transfection assays were conducted following the instructions of Lipofectamine^TM^ 2000. The MBNL1-AS1 expression suppressor plasmid (si-MBNL1-AS1 group)/negative control plasmid (si-NC group), miR-424-5p mimics/negative sequence (miR-NC group), miR-424-5p inhibitor and negative control (inhibitor NC group), and full length of SMAD7 (SMAD7 ov group) were transfected into AGS cell line. The MBNL1-AS1 overexpression plasmid (MBNL1-AS1 ov group)/control group (NC group) was transfected into the HGC-27 cell line.

### 2.4 Quantitative reverse transcriptase-polymerase chain reaction (qRT-PCR)

Total RNA was extracted from gastric cancer tissues and cell lines using the TRIzol reagent. The RNA was then reverse-transcribed into cDNA. The expression of lncRNA MBNL1-AS1 and SMAD7 was detected by ABI 7900HT RealTime PCR System using SYBR Green assays with GAPDH as the internal control. The expression of miR-424-5p was measured using TaqMan MicroRNA Assays with U6 as the internal control. The relative expression was calculated via the 2^−ΔΔCt^ method.

### 2.5 CCK-8 assay for cell proliferation

The cells (in good condition) were seeded into a 96-well plate until the cells were completely attached to the wall. A 20 μL CCK-8 reagent was then added to each well. The cells were cultured at 37°C for 2 h, then detected using a microplate reader. The remaining wells were cultured for 24, 48, 72, and 96 h with 20 μL CCK-8 reagent in an incubator with 5% CO_2_ at 37°C for 2 h. The absorbance value was determined using a microplate reader at 450 nm.

### 2.6 BrdU assay

The cells were incubated with BrdU (10 μM) for 30–60 minutes. Frozen and precipitated cells were suspended at 4°C and chilled with 70% V/V ethanol for at least 30 minutes (for up to seven days after treatment). The supernatant was washed once with PBS and incubated with freshly prepared 2 M HCl at room temperature for 30 minutes (mixed occasionally). The cells were washed twice with PBS and then re-suspended with PBS- Twain (containing 0.1% BSA and 0.2% Tween 20, pH 7.4). The anti-BrdU monoclonal antibody was added to the cell suspension then incubated in the dark at room temperature for 20 minutes. The sample was then washed twice with PBS- Twain. The cells were incubated with RNAse (50 μL, 100 mg/mL) at room temperature or 37°C for 15 minutes. FCM analysis was used to detect cell proliferation ability.

### 2.7 Colony formation assay

Cell suspension was prepared by gathering cells from each group during the logarithmic growth period. The cell suspension was diluted in gradient multiples and inoculated in culture medium dishes for three weeks. The culture was terminated when visible clones appeared in the petri dish. The supernatant was discarded, then the sample was fixed with 4% paraformaldehyde, and dyed with GIMSA staining solution for 10–30 minutes.

### 2.8 Flow cytometry assay for apoptosis

The cells (in good condition) were selected after 48 h of transfection, then aspirated. The supernatant was discarded, and the cells were washed twice with PBS, then aspirated again. The PBS solution was discarded, then 1 ~ 2 mL trypsin was added to the sample to digest cells, making them detached. The cells were gently mixed to form a cell suspension. A buffer was added to terminate the digestion. A 5 μL each of AnnexinV-FITC and PI were then added, gently shaken to mix, then incubated at 25°C, while avoiding light for 15 minutes. The rate of cell apoptosis was detected using a flow cytometer. Each group had three duplicate holes, and the experiment was repeated thrice.

### 2.9 Transwell assays

Cell migration assay: A 600 μL of RPMI-1640 complete culture medium containing fetal bovine serum and 200 μL of cell suspension were added to the lower and upper chambers of the transwell chamber and placed in a cell incubator for 24 h. The culture medium was removed, then the upper cells were gently wiped off using a cotton swab. The cells were washed with PBS, fixed with 4% paraformaldehyde for 30 minutes, and stained with 0.1% crystal violet for 10 minutes. A microscope was then used to visualize and count the number of cells stained with crystal violet.

Cell invasion assay: A 60 μL matrigel was mixed with 300 μL RPMI-1640 culture medium without serum. About 100 μL of the mixture was then spread in the upper chamber, as described in the cell migration assay.

### 2.10 Wound healing assay

The cells were collected into a 6-well plate (6 × 10^5^ cells per well). A 200 μL pipette head was used to scratch a line when the cells reached 100% confluence. The culture plates were washed with PBS to remove the cell fragments. The cells were cultured in an incomplete RPMI 1640 medium without FBS at 37°C. The initial wound was visualized using a light microscope, and the recovery area was cultured for 48 h. The wound healing area was calculated. Each experiment was conducted thrice.

### 2.10 RNA immunoprecipitation

Magna RIP RNA-Binding Protein Immunoprecipitation Kit was used for RIP assay. To detect the miRNA binding to LncRNA, AGS cells were lysed in RIP lysis buffer and incubated with Biotin-coupled probe of MBNL1-AS1 or olige probe which was pre-bound on magnetic beads. The purified miRNA was then subjected to qRT-PCR. To detect the LncRNA binding to miRNA, Biotin-coupled probe of miR-424-5p or Biotin-NC was processed through the same Protocol.

### 2.11 Animal experiments

The 6-week-old BALB/c nude mice were raised in a laboratory standard condition. Animal experiments were conducted following the Care and Use of Laboratory Animals and were approved by Lihuili Hospital. Mice were subcutaneously injected with GC cells and monitored daily to establish a subcutaneous tumor model. The tumor size was recorded every three days. The average tumor volume was calculated using the following formula: volume (mm^3^) = length × width^2^ × 0.5. The mice were sacrificed after three weeks of injection to measure tumor weight.

### 2.12 Dual-luciferase assay

The wild type and mutant luciferase expression vectors pGL3-MBNL1-AS1-wt and pGL3- MBNL1-AS1-mut of lncRNA MBNL1-AS1, or pGL3-SMAD7-wt and pGL3- SMAD7-mut of SMAD7 were transfected into AGS cells with miR-424-5p mimics and corresponding negative control miR-NC using Lipofectamine^TM^ 2000. Results were obtained after 48 h using the guideline of luciferase activity detection.

### 2.13 Western blot

The total protein was extracted using RIPA protein lysis buffer. The BCA kit was used for protein quantification. The sample was blocked with 5% skim milk at 25°C for 90 minutes. The samples were first incubated with the corresponding primary antibodies (Smad7, p-smad2, Smad2, p-smad3, Smad3, Bax, Vimentin, Cyclin D1, and GAPDH) at 4°C overnight. The samples were washed thrice with PBS (5 minutes each wash), then incubated with secondary antibody (1:1200) at 25°C for 2 h. The samples were then washed thrice with PBS (10 minutes each wash), then stained with ECL luminescent solution in a dark room and imaged using ChemiDocXRS+ system.

### 2.14 Statistical analyses

SPSS 20.0 software and GraphPad 7 were used for all statistical analyses. Data were expressed as mean ± standard deviation ($\overset{ˉ}{x}$± s). T-test was used for comparison between the two groups. Comparison between groups was conducted using one-way analyses of variance. Kaplan-Meier and log-rank analysis were used for survival analysis. P < 0.05 indicated statistically significant difference.

## 3. Results

### 3.1 lncRNA MBNL1-AS1 level in GC tissues and cells

**Figure 1.**
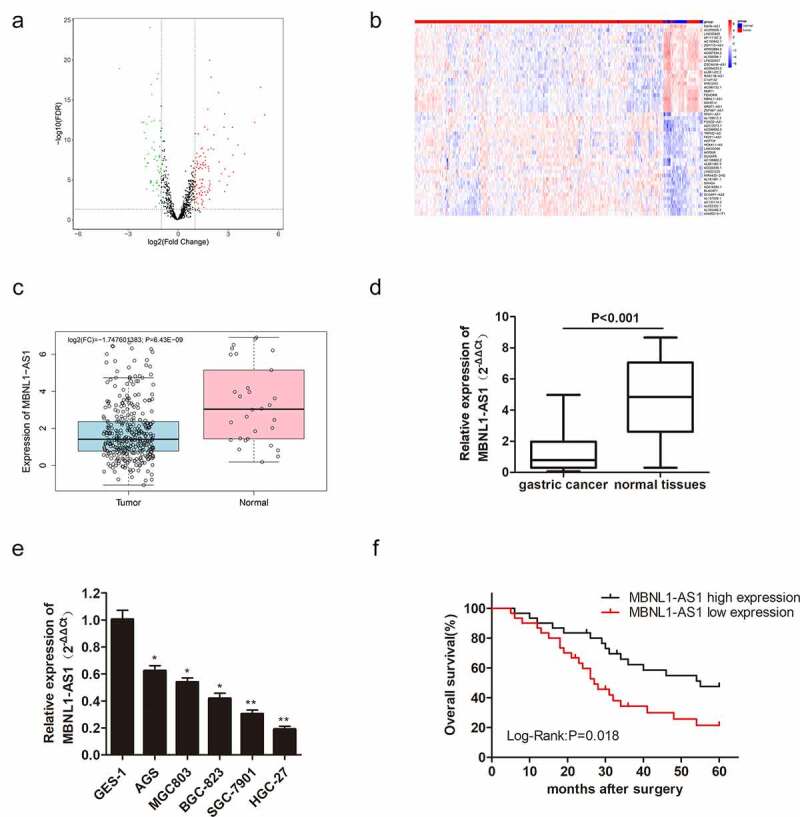
LncRNA MBNL1-AS1 level in gastric cancer tissues and cells, and its association with overall survival rate in GC patients. (a) Screened lncRNAs with varying expression in GC based on TCGA database. (b) Top 50 lncRNAs with differential expression in GC. (c) MBNL1-AS1 expression level in GC tissues based on TCGA database. (d) The expression of MBNL1-AS1 in 60 pairs of gastric cancer patient tissues and normal tissues. (e) The expression level of MBNL1-AS1 in five GC cell lines (AGS, MGC803, BGC-823, SGC-7901, HGC-27) and human gastric mucosal epithelial cell line GES-1. (f) The correlation between MBNL1-AS1 expression level and overall survival rate analyzed using Kaplan-Meier analysis.

As mentioned earlier, MBNL1-AS1 expression is decreased in some cancers. In this research, we utilized the TCGA database to screen the differentially expressed lncRNAs in GC (Figure 1(a)). The top 50 lncRNAs with abnormal up-regulations or down-regulations in GC are shown in Figure 1(b). Bioinformatics analysis in TCGA showed that MBNL1-AS1 was down-regulated in GC tissues compared with adjacent tissues (Figure 1(c)). qRT-PCR also showed that MBNL1-AS1 was slightly expressed in cancer tissues compared with normal tissues (P < 0.001, Figure 1(d)). MBNL1-AS1 was down-regulated in five GC cell lines (AGS, MGC803, BGC-823, SGC-7901, and HGC-27) compared with human gastric mucosal epithelial cell line GES-1 (Figure 1(e)). MBNL1-AS1 level was lowest in the HGC-27 cell line and highest in the AGS cell line. Subsequently, Kaplan-Meier analysis was used to assess the correlation between MBNL1-AS1 expression and the survival rate of GC patients. Patients with higher MBNL1-AS1 levels had higher overall survival rates (Figure 1(f)). All the evidence above demonstrates that MBNL1-AS1 is down-regulated in GC tissues and cells, positively related to the GC survival rate.

### 3.2 *LncRNA MBNL1-AS1 promotes cell apoptosis* in vitro *by inhibiting GC cell proliferation, migration, and invasion*

**Figure 2.**
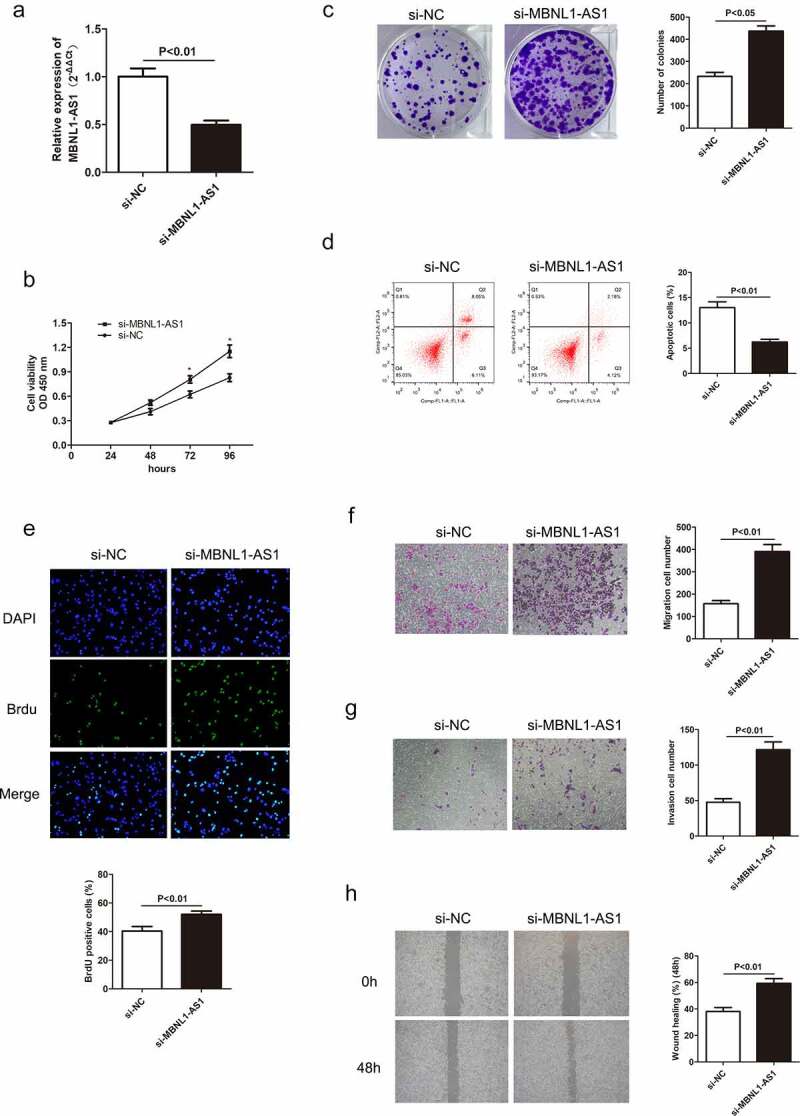
Knockdown of MBNL1-AS1 inhibits cell apoptosis by accelerating AGS cell proliferation, migration, and invasion. (a) The expression level of MBNL1-AS1 in AGS cells after transfection with si-MBNL1-AS1 or si-NC vectors. (b) CCK-8 assay showing cell viability in AGS cells after transfection with si-MBNL1-AS1 or si-NC. (c) Effects of MBNL1-AS1 knockdown on colony formation. (d) Flow cytometry analysis showing AGS cell apoptosis ability. (e) BrdU assay evaluating cell proliferation capacity in AGS cells transfected with si-MBNL1-AS1 or si-NC. (f-g) Transwell assays showing the effect of MBNL1-AS1 knockdown on cell migration and invasion. (h) Wound healing assay showing the migration ability in AGS.

**Figure 3.**
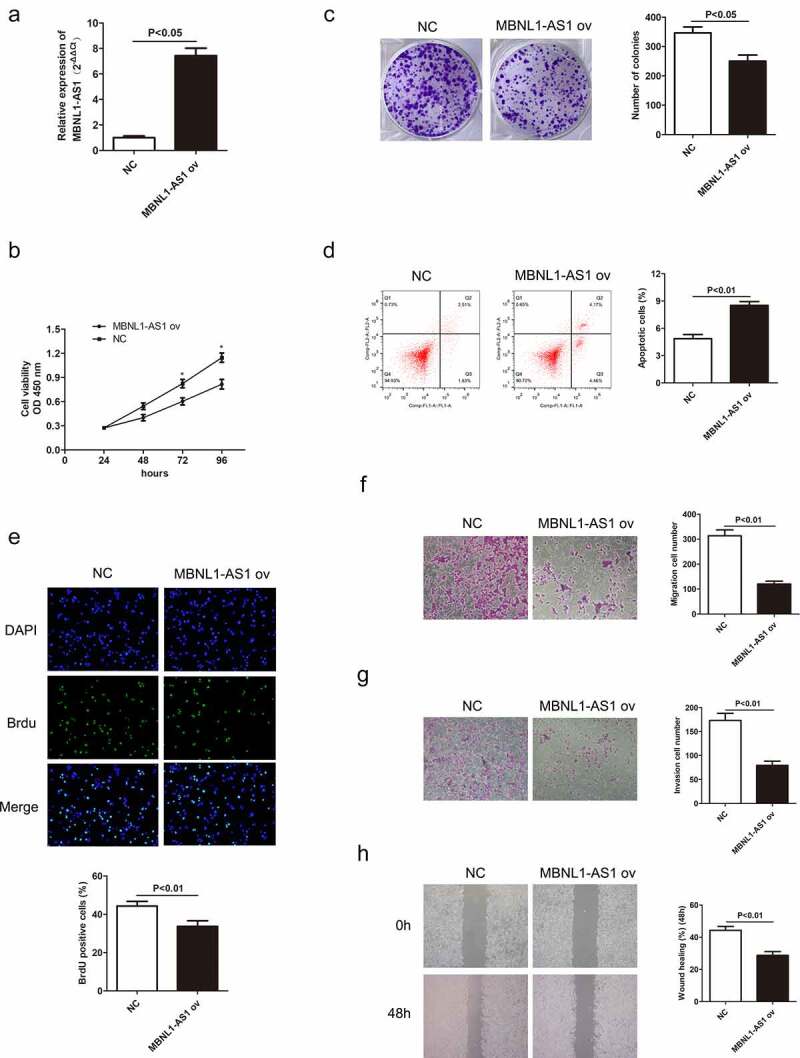
Overexpression of MBNL1-AS1 promotes cell apoptosis by inhibiting HGC-27 cell proliferation, migration, and invasion. (a) The expression level of MBNL1-AS1 in HGC-27 cells after transfection with MBNL1-AS1 ov or NC vectors. (b) CCK-8 assay showing cell viability in HGC-27 cells after transfection with MBNL1-AS1 ov or NC vectors. (c) Effects of increased MBNL1-AS1 level on colony formation. (d) Flow cytometry showing HGC-27 cell apoptosis ability in MBNL1-AS1 ov or NC group. (e) BrdU assay examining cell proliferation capacity of HGC-27 cells in MBNL1-AS1 ov or NC group. (f-g) Transwell assays showing the effect of MBNL1-AS1 up-regulation on cell migration and invasion. (h) Wound healing assay showing HGC-27 cell migration ability.

Decreased MBNL1-AS1 expression in GC tissues indicates its possible tumor-suppressor role in GC. To verify this hypothesis, we established a knockdown and overexpression system of MBNL1-AS1 in AGS and HGC-27 cell lines, respectively. We transfected AGS cells with blank plasmid and MBNL1-AS1 interference plasmid to establish si-NC and si-MBNL1-AS1 groups, respectively. The pcDNA3.1-MBNL1-AS1 was transfected into HGC-27 cells to obtain MBNL1-AS1 overexpression system (MBNL1-AS1 ov group) with NC group as the control. qRT-PCR was applied to determine MBNL1-AS1 expression level. MBNL1-AS1 level significantly decreased in the si-MBNL1-AS1 group compared with the si-NC group (P < 0.01) (Figure 2(a)). Meanwhile, MBNL1-AS1 level was higher in the MBNL1-AS1 ov group than in the NC group (P < 0.05, Figure 3(a)). CCK-8 analysis was used to assess cell proliferation. Knockdown of MBNL1-AS1 enhanced cell viability in AGS cell line compared with the si-NC group (Figure 2(b)), while MBNL1-AS1 overexpression inhibited HGC-27 cell viability (Figure 3(b)). Colony formation assay and BrdU assay showed that MBNL1-AS1 silencing enhanced the proliferative capacity of AGS cell line in the si-MBNL1-AS1 group compared with the si-NC group (Figures 2(c,e)). Nevertheless, MBNL1-AS1 overexpression reduced the colony formation and cell proliferative capacity in the HGC-27 cell line (Figures 3(c,e)). Furthermore, GC cell apoptosis ability was evaluated using a flow cytometry experiment. Knockdown of MBNL1-AS1 restrained AGS cell apoptosis (P < 0.01) (Figure 2(d)). Conversely, MBNL1-AS1 overexpression significantly increased HGC-27 cell apoptosis (P < 0.01, Figure 3(d)). These results indicate that MBNL1-AS1 can inhibit GC cell proliferation and promote cell apoptosis. Transwell and wound healing assays were used to determine cell metastasis ability. Transwell assays showed that the cell migration and invasion abilities increased in AGS cells transfected with si-MBNL1-AS1 compared with the si-NC group (P < 0.01, Figures 2(f) and 2 G). The overexpression of MBNL1-AS1 restrained the migration and invasion of HGC-27 cells (P < 0.01, Figures 3(f) and 3 G). Wound healing assay further demonstrated that MBNL1-AS1 knockdown accelerated AGS cell migration (P < 0.01, Figure 2(h)) while MBNL1-AS1 overexpression remarkably inhibited migratory abilities of HGC-27 cells (P < 0.01, Figure 3(h)). Taken together, these findings indicate that MBNL1-AS1 expression is negatively correlated with GC cell proliferation and invasion, and thus can act as a tumor suppressor.

### 3.3 *LncRNA MBNL1-AS1 inhibits GC tumor growth* in vivo

**Figure 4.**
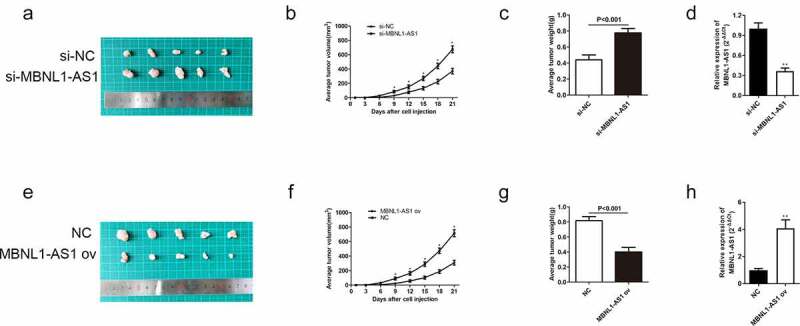
MBNL1-AS1 restrains GC tumor growth *in vivo*. (a) The image of xenograft tumor after treatment with si-MBNL1-AS1 or si-NC. (b) The average tumor volume (mm^3^) (measured every three days) in si-MBNL1-AS1 and si-NC groups after a 3-week injection. (c) The tumor weight (g) in si-MBNL1-AS1 and si-NC groups after a 3-week injection. (d) qRT-PCR assay for MBNL1-AS1 expression in AGS cells that were transfected with si-MBNL1-AS1 and si-NC among xenograft tumors. (e)The image of xenograft tumor after treatment with MBNL1-AS1 ov or NC group. (f) The average tumor volume (mm^3^) (measured every three days) in MBNL1-AS1 ov and NC groups after a 3-week injection. (G) The tumor weight (g) in MBNL1-AS1 ov and NC groups after a 3-week injection. (h)qRT-PCR assay for MBNL1-AS1 expression in HGC-27 cells that were transfected with MBNL1-AS1 ov and NC among xenograft tumors.

Herein, we also further elucidated the role of MBNL1-AS1 on xenograft tumor *in vivo*. The AGS cells transfected with si-MBNL1-AS1 or si-NC plasmids and HGC-27 cells transfected with MBNL1-AS1 ov or NC plasmids were injected in nude mice to determine their effect *in vivo*. The tumor size was recorded every three days during the 3-week injection period. The tumor volume growth was faster in the si-MBNL1-AS1 group (MBNL1-AS1 knockdown) than in the control group (Figures 4(a,b)). In contrast, the tumor volume growth was slower in the MBNL1-AS1 ov-transfected group than in the NC group (Figures 4(e) and 4 F). The average tumor weight showed the same trend. For instance, the average tumor weight was higher in the MBNL1-AS1 knockdown group than in the control group (P < 0.001, Figure 4(c)). Overexpression of MBNL1-AS1 significantly alleviated the average tumor weight difference (P < 0.001, Figure 4(g)). Then, we detected expression of MBNL1-AS1 in xenograft tumors through the use of qRT-PCR assays. We found that si-MBNL1-AS1 group had lower expression of MBNL1-AS1 compared to the si-NC group (Figure 4(d)), while MBNL1-AS1 ov group had higher expression of MBNL1-AS1 than the NC group (Figure 4(h)). Similar to *in vitro* analysis, MBNL1-AS1 played a positive role in restraining GC tumor growth *in vivo*.

### 3.4 LncRNA MBNL1-AS1-targeted miR-424-5p directly and negatively regulates miR-424-5p

**Figure 5.**
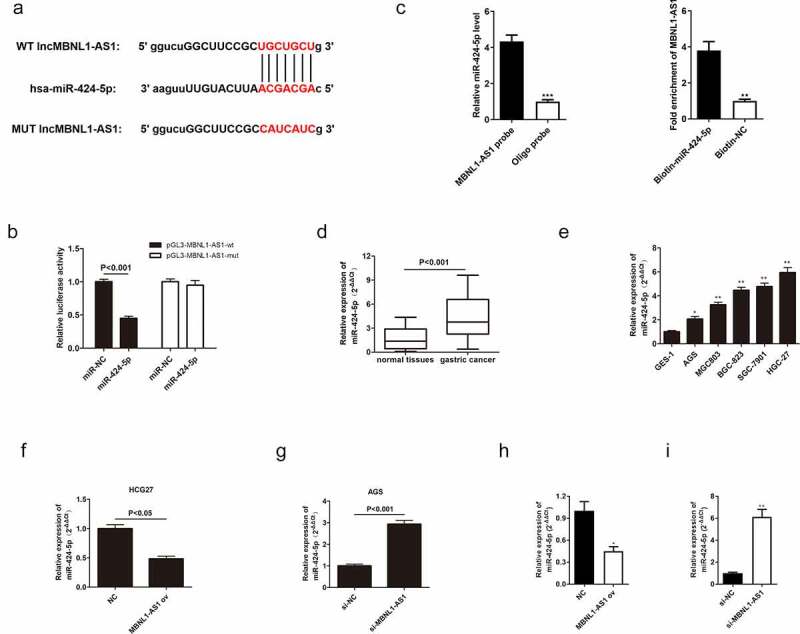
MBNL1-AS1 directly targets and negatively regulates miR-424-5p. (a) The potential binding sites between MBNL1-AS1 and miR-424-5p. (b) Dual-luciferase assay showing the direct interaction between MBNL1-AS1 and miR-424-5p. (c) RIP experiment showed that MBNL1-AS1 directly targeted miR424-5p. (d) The expression level of miR-424-5p in 60 pairs of gastric cancer patient tissues and normal tissues. (e) The expression level of miR-424-5p in five gastric cancer cell lines (AGS, MGC803, BGC-823, SGC-7901, HGC-27) and human gastric mucosal epithelial cell line GES-1. (f) qRT-PCR assay examining miR-424-5p level in HGC-27 cells transfected with MBNL1-AS1 ov or NC. (g) qRT-PCR assay examining miR-424-5p level in AGS cells after transfection with si-MBNL1-AS1 or si-NC vectors. (h) qRT-PCR assay for miR-424-5p expression in HGC-27 cells that were transfected with MBNL1-AS1 ov and NC among xenograft tumors. (i) qRT-PCR assay for miR-424-5p expression in AGS cells that were transfected with si-MBNL1-AS1 and si-NC among xenograft tumors.

To explore the mechanism of MBNL1-AS1 in GC cell progression, its downstream target needed to be found. Previous studies have shown that lncRNAs bind to miRNAs, thus serving as ceRNA to regulate cell function [22,23]. According to the intersection of possible targets predicted by miRanda, LncBase, and Starbase databases, miR-424-5p contained potential binding sites with MBNL1-AS1 (Figure 5(a)). The following dual luciferase assay further proved that MBNL1-AS1 is directly correlated with miR-424-5p. In the cell experiment transfected with wild-type vector pGL3-MBNL1-AS1-wt, the luciferase activity was significantly reduced in miR-424-5p group compared with the control group. However, luciferase activity was not statistically different between the miR-424-5p group transfected with mutant vector pGL3- MBNL1-AS1-mut and the control group (Figure 5(b)). RIP assay revealed that there was specific bind locations of MBNL1-AS1 on miR-424-5p (Figure 5(c)). qRT-PCR was used to detect miR-424-5p expression level in GC tumor tissues and cells. The miR-424-5p was distinctly up-regulated in patient tissues (P < 0.001, Figure 5(d)). For instance, miR-424-5p was up-regulated in GC cell lines (AGS, MGC803, BGC-823, SGC-7901, HGC-27) compared with normal cell line GES-1(P < 0.001, Figure 5(e)). These results show that miR-424-5p is overexpressed in gastric tumor tissues and cancer cells. Moreover, the relationship between miR-424-5p and MBNL1-AS1 was assessed by measuring miR-424-5p level in MBNL1-AS1 knockdown system and MBNL1-AS1 overexpression system. The miR-424-5p level was reduced in the HGC-27 cell line transfected with MBNL1-AS1 ov compared with the control group (P < 0.05, Figure 5(f)). In contrast, the miR-424-5p level was elevated in the AGS cell line transfected with si-MBNL1-AS1 (P < 0.05, Figure 5(g)). Lastly, we detected expression of miR-424-5p in xenograft tumors through the use of qRT-PCR assays. We found that MBNL1-AS1 ov group had lower expression of miR-424-5p than the NC group (Figure 5(h)), while si-MBNL1-AS1 group had higher expression of miR-424-5p compared to the si-NC group (Figure 5(i)). These results show that MBNL1-AS1 directly binds to miR-424-5p and is negatively associated with the miR-424-5p level.

### 3.5 miR-424-5p knockdown alters GC cell progression mediated by si-MBNL1-AS1

**Figure 6.**
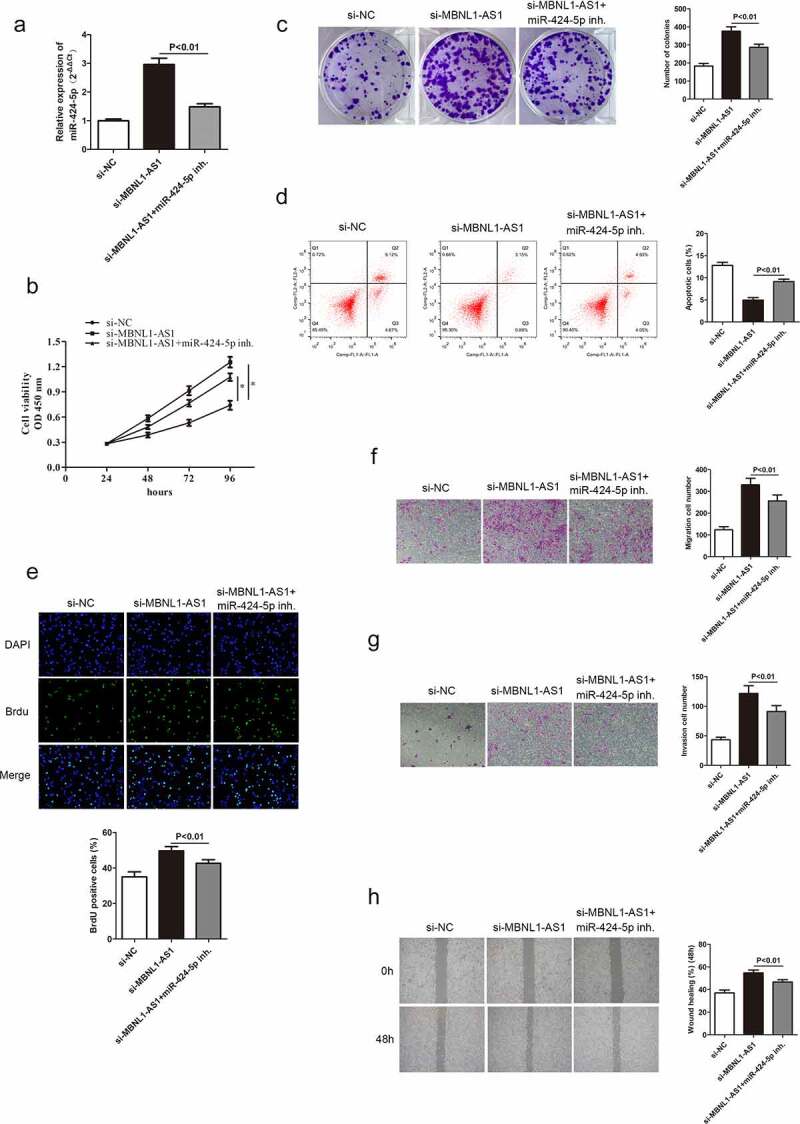
Knockdown of miR-424-5p reverses the promotion of GC cell progression mediated by si-MBNL1-AS1. (a) qRT-PCR detecting miR-424-5p expression level in three groups: si-NC, si-MBNL1-AS1, and si-MBNL1-AS1+ miR-424-5p inhibitor. (B, C, and E) CCK-8, colony formation, and BrdU assays showing cell proliferation. (d) Flow cytometry analysis showing cell apoptosis capacity. (F, G, and H) Transwell and wound healing assays showing the effect of cell migration and invasion abilities among three groups.

A previous study confirmed that MBNL1-AS1 inhibits GC cell progression and directly regulates miR-424-5p. Herein, rescue experiments were conducted to detect the miR-424-5p effect on GC cells to illustrate whether MBNL1-AS1 exerts its biological function through miR-424-5p. miR-424-5p was poorly expressed in the si-MBNL1-AS1 group with a miR-424-5p inhibitor in GC cell line (AGS) compared with the si-MBNL1-AS1 group (Figure 6(a)). The GC cell proliferation, apoptosis, migration, and invasion abilities in three groups (si-NC, si-MBNL1-AS1, si-MBNL1-AS1 + miR-424-5p inhibitor) are shown in Figure 6(b-h). Previous findings showed that MBNL1-AS1 knockdown could promote GC cell progression in the si-MBNL1-AS1 group (Figure 2(b-h)). We further investigated the regulatory correlation between miR-424-5p and MBNL1-AS1 by simultaneously giving miR-424-5p inhibitor to si-MBNL1-transfected group. CCK-8, colony formation, and BrdU assays showed that miR-424-5p inhibitor restrained the GC cell proliferation, which was enhanced by si-MBNL1 (P < 0.01, Figures 6(b), 6C, 6E), indirectly indicating that the down-regulation of miR-424-5p could reverse the biological function of si-MBLN1-AS1. Flow cytometry experiment showed that down-regulation of miR-424-5p promoted GC cell apoptosis compared with the si-MBNL1-AS1 group (P < 0.01, Figure 6(d)). Transwell and wound healing assays indicated that reduced miR-424-5p level alleviated GC cell migration and invasion capacities compared with the si-MBNL1-AS1 group (P < 0.01, Figure 6(f-h)). These results show that decreased miR-424-5p level inhibits GC cell progression caused by MBNL1-AS1 silencing, indicating that miR-424-5p can alleviate the MBNL1-AS1 effect on GC.

### 3.6 MiR-424-5p directly interacts with Smad7

**Figure 7.**
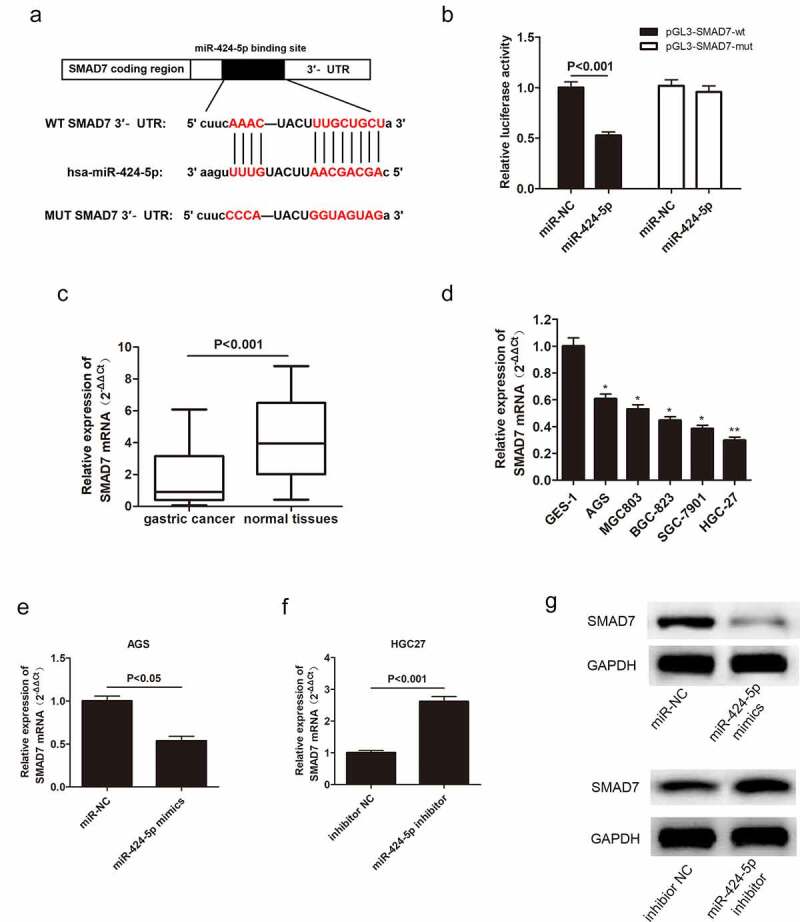
Smad7 targets miR-424-5p via negative correlation. (a) Bioinformatics databases showing that Smad7 directly binds with miR-424-5p. (b) Dual-luciferase assay showing that miR-424-5p directly interacts with Smad7. (c) qRT-PCR showing the expression level of Smad7 mRNA in 60 pairs of GC patient tissues. (d) The expression level of Smad7 mRNA in five GC cell lines (AGS, MGC803, BGC-823, SGC-7901, HGC-27) and human gastric mucosal epithelial cell line GES-1. (e) QRT-PCR assay examining Smad7 mRNA level in AGS cells transfected with mimics-NC or miR-424-5p mimics. (f) qRT-PCR assay showing Smad7 mRNA level in HGC-27 cells transfected with inhibitor NC or miR-424-5p inhibitor. (g) Western blot showing the expression level of Smad7 in mimics NC and miR-424-5p mimics, inhibitor NC and miR-424-5p inhibitor.

In this study, the possible mechanism of miR-424-5p-regulated GC cell proliferation and invasion was also clarified. The bioinformatics databases showed that miR-424-5p had a potential binding site with Smad7, a type of inhibitory protein (Figure 7(a)). Dual-luciferase assay provided evidence supporting the direct relationship between miR-424-5p and Smad7 (Figure 7(b)). qRT-PCR was adopted to examine Smad7 mRNA expression level in GC tissues from 60 patients and five GC cell lines. Smad7 mRNA was down-regulated in both GC tissues and cells compared with the normal groups (Figure 7(c-d)). Moreover, we obtained miR-424-5p overexpression and inhibition groups by introducing miR-424-5p mimics and miR-424-5p inhibitor to AGS and HGC-27 cell lines, respectively, to illustrate the relationship between miR-424-5p and Smad7. Smad7 mRNA level reduced in the overexpression group while increasing in the knockdown group (P < 0.05, Figure 7(e-f)). Similarly, the Western blot showed a similar trend of Smad7 expression level. Moreover, Smad7 was downregulated in the overexpression group while up-regulated in the knockdown group. (Figure 7(g)). These results show that miR-424-5p can directly bind to and negatively regulate Smad7 expression.

### 3.7 Smad7 reverses the promotion of GC cell progression caused by miR-424-5p

**Figure 8.**
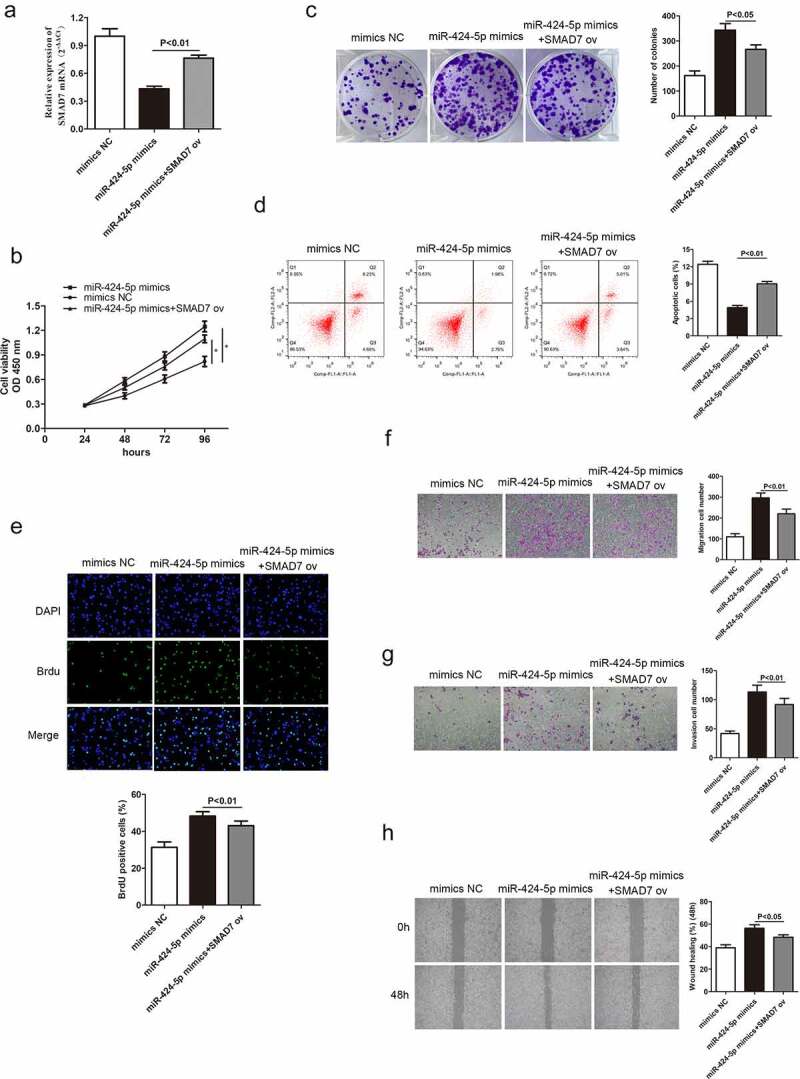
Smad7 reverses the promotion of GC cell progression caused by miR-424-5p. (a) qRT-PCR detecting Smad7 mRNA level in three groups: mimics-NC, miR-424-5p mimics, and miR-424-5p mimics + Smad7 ov. (B, C, and E) CCK-8, colony formation, and BrdU assays showing cell proliferation ability. (d) Flow cytometry showing cell apoptosis ability. (F, G, and H) Transwell and wound healing assays showing cell migration and invasion abilities among the three groups. *P < 0.05.

Based on the above conclusions, we hypothesized that Smad7 might alter miR-424-5p and MBNL1-AS1 function in GC cells. miR-424-5p mimics were introduced in AGS cells to obtain miR-424-5p overexpression group to test the hypothesis. qRT-PCR indicated that Smad7 mRNA level was up-regulated in the overexpression group (P < 0.01, Figure 8(a)). Rescue experiments, including CCK-8, colony formation and BrdU assays, were used to describe cell proliferation ability. The overexpression of Smad7 deteriorated GC cell growth compared with the miR-424-5p mimics group (Figures 8(b), 8C, 8E). Flow cytometry showed that increased Smad7 mRNA level enhanced cell apoptosis (P < 0.01, Figure 8(d)). Transwell and wound healing assays showed that the up-regulation of Smad7 mRNA level impaired migration and invasion abilities in GC cells compared with the miR-424-5p mimics group (Figure 8(f-h)). These results show that the up-regulation of Smad7 level can inhibit GC cell growth, migration and invasion, contrary to the effects of miR-424-5p overexpression.

### 3.8 MBNL1-AS1 affects TGF-β/SMAD pathways by regulating Smad7

**Figure 9.**
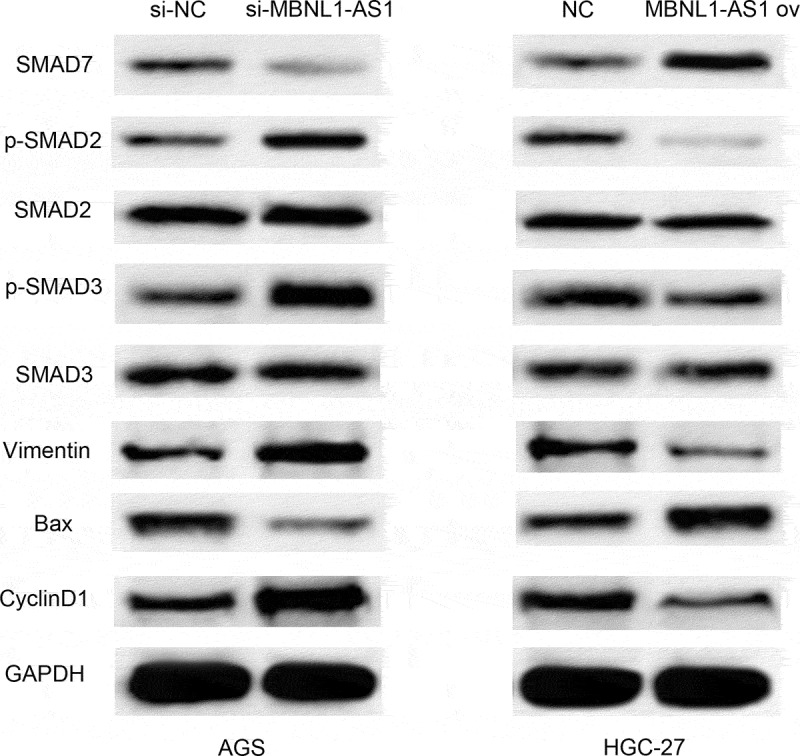
MBNL1-AS1 affects TGF-β/SMAD pathways by regulating Smad7. Western blot showing the correlated protein levels of TGF-β pathway markers (p-Smad2, p-Smad3, Smad2, and Smad3), apoptosis-related protein Bax, cyclin pathway-related protein Cyclin D1, and EMT marker Vimentin in GC with MBNL1-AS1 knockdown or overexpression.

So far, we have confirmed that MBNL1-AS1 inhibits GC cell progression. TGF-β/EMT pathway is closely related to cancer promotion [24]. Moreover, Smad7 was identified as a target of miR-424-5p and MBNL1-AS1, which negatively regulates miR-424-5p. Smad7, induced by all members of the TGF-β superfamily, can inhibit the TGF-β signaling pathway at multiple levels. It can also negatively regulate TGF-β signaling pathway [25]. Therefore, MBNL1-AS1 may affect TGF-β/SMAD pathways by interacting with Smad7. Western blot was applied to detect the expression of TGF-β pathway-correlated proteins (p-Smad2, p-Smad3) and EMT pathway-correlated protein Vimentin. Knockdown of MBNL1-AS1 in si-MBNL1-AS1 group downregulated Smad7 while it up-regulated p-Smad2 and p-Smad3 in TGF-β pathway (Figure 9). Higher level of Vimentin was also detected in si-MBNL1-AS1 group. Conversely, high level of MBNL1-AS1 was correlated with high Smad7 level and low level of p-Smad2 and p- Smad3 in the TGF-β pathway. Moreover, low level of Vimentin was detected in MBNL1-AS1 ov group compared with the control. Moreover, knockdown of MBNL1-AS1 up-regulated cyclin pathway-related protein Cyclin D1 (Figure 9). In contrast, upregulation of MBNL1-AS1 decreased Cyclin D1 expression. Similarly, our previous results showed that the silencing of MBNL1 can alleviate the apoptosis rate of GC cells. The analyses of apoptosis-related protein Bax supported the apoptosis suppression effect of si-MBNL1. Western blot showed that Bax was significantly down-regulated in the si-MBNL1-transfected group (Figure 9). This result demonstrates that MBNL1 influences GC cell apoptosis by mediating the expression of apoptosis-related protein Bax. Consequently, MBNL1-AS1 affects TGF-β/SMAD pathways by regulating Smad7 expression involved in GC cell progression.

## 4. Discussion

Over the past years, the theory about the interactions between lncRNAs and miRNAs has been widely accepted [26]. Prior studies have shown that lncRNA MBNL1-AS1 is involved in the progression of diverse tumors and thus can act as an effective tumor indicator for cancer cell life cycle and metastasis. For instance, MBNL1-AS1 expression is decreased in NSCLC and suppresses NSCLC cell promotion by sponging miR-135a-5p [27]. In bladder cancer tissues, MBNL1-AS1 is down-regulated. Overexpression of MBNL1-AS1 can inhibit tumorigenesis via miR-135a-5p/PHLPP2/FOXO1 axis [28]. MBNL1-AS1 also was reported that promoting the progression of acute myocardial infarction [29]. Gastric cancer (GC) has been identified as the most common malignant tumor for a long time. However, the function of MBNL1-AS1 in GC is rarely investigated, and thus its possible mechanism should be further explored. Moreover, there is little lucubration about the exact biological function of MBNL1-AS1 in GC. This study aimed to reveal the specific molecular mechanism underlying mediation and regulation of GC development, and thus may provide a novel strategy for GC regimens and ameliorate prognosis.

Herein, qRT-PCR showed that MBNL1-AS1 was down-regulated in GC tissues and cell lines (Fig.1), possibly suggesting that aberrant down-regulation of MBNL1-AS1 is associated with the course of GC. Similarly, the overall survival prediction exhibited that extended lifespan is positively related to the high expression level of MBNL1-AS1. Thereafter, the alteration after knockdown of MBNL1-AS1 in GC cells using si-MBNL1-AS1 was determined using CCK-8 assay, BrdU assay, colony-forming assay, transwell assay, and wound healing assay. Results showed that silencing of MBNL1-AS1 accelerated GC cell proliferation by restraining cell apoptosis and potentiated cell migration and invasion (Fig.2), indicating the tumor-suppressing effect of MBNL1-AS1. However, overexpression of MBNL1-AS1 significantly reduced cell proliferation, migration, and invasion *in vitro* and *in vivo* (Figs.3 and 4), verifying that MBNL1-AS1 can function as a tumor suppressor in GC. Moreover, miR-424-5p was identified as a potential target gene of MBNL1-AS1. Dual-luciferase assay verified the interaction of these two factors (Figure 5). qRT-PCR confirmed that MBNL1-AS1 can bind to miR-424-5p via negative modulation. Furthermore, rescue experiments demonstrated that decreased miR-424-5p level could inhibit GC cell promotion caused by MBNL1-AS1 silencing (Figure 6).

Transforming growth factor-β (TGF-β) affects multifarious biological processes, including cell growth, metastatic, differentiation, apoptosis, and autophagy [30–33]. Mounting evidence in recent years has proven that the activation of TGF-β signaling pathway is pathologically correlated with various cancers [34–38]. The TGF-β pathway studies have shown that Smad7 can attenuate against TGF-β pathway, thereby inhibiting the signal-transducing proteins Smad2 and Smad3 [39,40]. In the present study, Smad7 was identified as a target of miR-424-5p. Western blot assay showed that up-regulation of MBNL1-AS1 significantly up-regulated Smad7, while it down-regulated p-Smad2 and p-Smad3. However, the upregulation did not significantly alter total Smad 2 and Smad3 expressions. These results suggest that MBNL1-AS1 can stimulate Smad7, thereby inhibiting TGF-β pathway (descent level of phosphorylation Smad2 and Smad3). MBNL1-AS1 knockdown showed the opposite results (Figure 9). A significant reduction of Vimentin was also observed in MBNL1-AS1 up-regulated group. Additionally, overexpression of MBNL1-AS1 up-regulated Bax and down-regulated CyclinD1, further confirming that MBNL1-AS1 hinders GC cell growth by promoting apoptosis. Collectively, these results show that MBNL1-AS1 activates TGF-β/EMT pathways by regulating miR-424-5p /Smad7 axis.

In summary, aberrant MBNL1-AS1 level was detected in GC tissues and cells. Specifically, increased MBNL1-AS1 inhibited GC cell proliferation, migration, and invasion via TGF-β/EMT pathways by modulating miR-424-5p/Smad7 axis. These findings strongly indicate that MBNL1-AS1 is a crucial tumor suppressor in GC. Therefore, MBNL1-AS1 and its downstream regulators miR-424-5p /Smad7 axis are promising targets for GC therapy. However, further mechanism should be investigated.

## 5. Conclusions

Taken together, these results illustrate that lncRNA MBNL1-AS1 inhibits GC progression by modulating miR-424-5p/ Smad7 axis, and it is associated with TGF-β/EMT pathways. This study might define an innovative and effective biomarker for GC diagnosis.
